# Neurodevelopmental outcomes are normal in congenital hypothyroid children diagnosed early and treated aggressively over the first three years

**DOI:** 10.1186/1687-9856-2013-S1-O23

**Published:** 2013-10-03

**Authors:** Ben Albert, Natasha Heather, Wayne Cutfield, Dianne Webster, Alistair Gunn, Craig Jefferies, Trecia Wouldes, Caitrin Roberts, Sheryl Tregurtha, Heather Stewart, Sarah Mathai, José Derraik, Paul Hofman

**Affiliations:** 1 Liggins Institute, Faculty of Medical and Health Sciences, University of Auckland, 1023 Auckland, New Zealand; 2 Department of Psychological Medicine, Faculty of Medical and Health Sciences, University of Auckland, 1023 Auckland, New Zealand; 3 Physiology, Faculty of Medical and Health Sciences, University of Auckland, 1023 Auckland, New Zealand; 4 National Testing Centre, LabPlus, Auckland District Health Board, 1148 Auckland, New Zealand; 5 Gravida: National Centre for Growth and Development, 1023 Auckland, New Zealand; 6 Starship Children’s Hospital, Auckland District Health Board, 1023 Auckland, New Zealand

## 

Despite marked improvements in developmental outcome with newborn screening and early levothyroxine replacement most follow up studies of congenital hypothyroidism (CH) show a persistent mild deficit in total IQ. It has been considered that these deficits in neurocognitive function occur *in utero* and thus even early therapy cannot completely normalise development.

Alternatively the deficits could be caused by delayed diagnosis and inadequate early treatment. In Auckland, early diagnosis occurs via our newborn screening programme along with an aggressive high dose treatment paradigm following referral to the endocrine service. Thus we hypothesised that early diagnosis and aggressive therapy with close monitoring would result in normal neurocognitive outcomes.

## Methods

A blinded prospective sibling-matched study was undertaken. Subjects were otherwise healthy children and adolescents aged 4 to 18 years. Exclusion criteria for both CH subjects and sibling controls included chronic illness, other congenital problems or documented developmental delay, cerebral palsy or other disability. Assessments included WPPSI for children under 7 years old, WISC IV for children > 7 years and several tests of motor function including the Berry assessment of visual motor function, PPVT and ABC. Auxological data were collected and body composition was assessed using DEXA scans.

49 children with CH and 53 sibling controls were recruited. Control subjects were younger (8.5 vs 10.4 years) but had similar gender proportions (54%female vs 60% female), height SDS (0.81 vs 0.84) and weight SDS (1.05 vs 0.97). In the CH group, 22% had athyreosis, 20% had dyshormonogenesis and 57% had ectopia. Average time to diagnosis was 12 ± 6.7 days and free T4 was normal by 16.6 ± 5.7 days. There was no difference in Verbal IQ between control and CH subjects (93.6 vs 96.7), Overall IQ (95.2 vs 95.1) although there was a trend to better processing speed in the control subjects (97.3 vs 95.1; p=0.07). There was no difference between groups for motor function although there was a trend to better overall ABC scores in the CH group (60.9 ± 29.8% vs 49.7 ± 29%; p=0.06). There were no differences in body composition between the two groups although BMD trended to being lower in the CH group (0.90 vs 0.98; p=0.067). There was no association with developmental outcomes and the age at diagnosis.

Conclusion, the current Auckland diagnosis and treatment paradigm results in neurocognitive outcomes no different to siblings. BMD was lower in the CH group, possibly suggesting the children have been mildly over treated.

